# Varicella zoster virus in bell's palsy: a prospective study

**DOI:** 10.1590/S1808-86942010000300016

**Published:** 2015-10-20

**Authors:** Mônica Alcantara de Oliveira Santos, Hélio H. Caiaffa Filho, Melissa Ferreira Vianna, Andressa Guimarães do Prado Almeida, Paulo Roberto Lazarini

**Affiliations:** 1 ENT; Graduate Student (MSc) – School of Medical Sciences – Santa Casa de São Paulo; 2 PhD; Professor; Technical Director of the Molecular Biology Lab Service – Central Lab – University Hospital – Medical School of the University of São Paulo (FM-USP); 3 MSc. PhD student – School of Medical Sciences Santa Casa de São Paulo; 4 MD; ENT; Former Resident from the ENT Department – Santa Casa de Misericordia de São Paulo; 5 PhD; Assistant Professor of Otorhynolaryngology – Santa Casa de Misericordia de São Paulo

**Keywords:** prospective studies, varicella, bell's palsy, facial paralysis, herpes zoster

## Abstract

Although Bell's palsy is the major cause of acute peripheral facial palsy, its pathogenesis remains unknown. Reactivation of the varicella zoster virus has been implicated as one of the main causes of Bell's palsy, however, studies which investigate the varicella zoster virus reactivation in Bell's palsy patients are mostly Japanese and, therefore, personal and geographic characteristics are quite different from our population.

**Aims:** To determine varicella zoster virus frequency in saliva samples from patients with Bell's palsy, using PCR.

**Material and Method:** One hundred seventy one patients with acute peripheral facial palsy were prospectively enrolled in this study. One hundred twenty were clinically diagnosed with Bell's palsy, within one week of onset of the disease and no previous anti-viral therapy. We had 20 healthy adults as controls. Three saliva samples were collected from patients and controls at initial examination and at one and two weeks later. The detection of the varicella zoster virus DNA was performed using PCR.

**Results:** Varicella zoster virus was detected in two patients (1.7%). The virus was not identified in saliva samples from the controls.

**Conclusions:** Varicella zoster virus was detected in 1.7% of saliva samples from patients with Bell's palsy, using PCR.

## INTRODUCTION

Peripheral Facial Palsy (PFP) was described by Sir Charles Bell (1774-1842) in 1821. Initially, all cases of facial nerve paralysis were called Bell's palsy (BP). Notwithstanding, after the causes of the disease were discovered, only the idiopathic cases were called Bell's palsy.

In studies about PFP, BP is the most common clinical presentation^1^, ^2^, ^3^. Its incidence was estimated in 20 to 30 cases for every 100,000 people^4^, ^5^, ^6^. Although it is the most frequent type of PFP, the cause of BP is still the object of many questions and theories.

The Varicella Zoster Virus (VZV) was one of the first viruses to be associated with the PFP. Back in 1907, James Ramsay Hunt described PFP manifestations and the typical skin lesions (vesicles and bubbles) on the skin of the ear conchae, frequently associated with tinnitus and vertigo. This clinical picture caused by the VZV started to be called Ramsay Hunt Syndrome (RHS), in homage to the author^7^.

Usually, the skin lesions crop up before the PFP; nonetheless, in 14% of the cases this happens afterwards, and may be absent in some individuals^8^. These cases of VZV-caused PFP and without skin manifestation have been called zoster sine herpete (ZSH).

In 1991, Dlugosch^9^ et al. described the VZV detection technique through the polymerase chain reaction (PCR) test. Such technique drove new publications which confirmed the presence of the herpes virus in cases of PFP, among them: Furuta et al.^10^, who used an oropharynx swab; Murakami et al.^11^, PCR in ear skin exudate; Pitkäranta et al.^12^, tear PCR; Furuta et al.^13^ and Lazarini et al.^14^, PCR in the saliva.

McCormick^15^ was the first to propose the theory of viral reactivation, according to which, after an initial infection the virus would pass through the blood current or the axon in a retrograde way, all the way to the sensitive ganglia and there they remain latent^16^, ^17^, ^18^. The viral reactivation happens through a reduction of the immune activity which could be triggered by metabolic alterations^15^, surgical or dental procedures^19^,^20^ or event under stress or immunesuppression^21^.

The viruses, when reactivated, replicate and spread through the facial nerve and its branches, causing the inflammatory process which culminates with the PFP. One of the facial nerve branches, the chorda tympani nerve responsible for innervating the submandibular and sublingual glands, would also receive these reactivated viruses and, when stimulating salivation, it would make possible for these agents to pass to the saliva. In the saliva it would be possible to identify the viral DNA by means of the PCR^14^.

Of course that knowing the causing agent and identifying the cause for the BP would guide us not only in our approach, but also regarding a better treatment for our patients with PFP. Unfortunately most of the papers which discuss the VZV prevalence in saliva as a biologic agent of BP, do it in regards of the Japanese population, which establishes a population group and geographic characteristics which are very different from those of our population.

In general, the prevalence of the herpes virus is not the same throughout the world, there are variations between countries^22^,^23^ and regions^24^. The very prevalence of PFP is also variable. In Madrid, the annual incidence was estimated to be 24.1 per 100 thousand people^25^. In Sicily^26^, the prevalence was 642.8 per 100 inhabitants, almost thirty times larger.

These epidemiological variations in viral manifestation can cause a different etiological profile for BP in our settings, and such fact has not been studied yet.

Based on this reality, it is important to study the relationship between BP and the varicella zoster virus, describing its frequency and characteristics in our settings. Thus, the goal of our paper was to study the frequency of the varicella zoster virus in the saliva of individuals with Bell's Palsy using PCR.

## MATERIALS AND METHODS

This was a prospective study carried out in a tertiary hospital, which was submitted and approved by the Ethics Committee for Research with Human Beings of the present institution (Project 080/06).

We prospectively assessed 120 patients seen in the Department of Otorhinolaryngology and clinically diagnosed as BP bearers, from August of 2002 and October of 2007, according with the following inclusion criteria:

a) Acute PFP;
b) Symptom onset of up to one week prior to the date of the first medical visit;
c) Clinical and otorhinolaryngological exam without evidence of other causal factors for the paralysis;
d) No treatment with antiviral drugs.

The control group was made up of 20 adults without otological, neurotological alterations or acute infections.

In the first visit, the patients were submitted to an interview and general physical and otorhinolaryngological exam. They were all referred to weekly evaluations in order to follow up the paralysis symptoms, which was measured according to the House-Brackmann scale^27^,^28^.

Upon admission, BP patients and controls were submitted to saliva collection from the mouth floor using a disposable 5ml syringe without needle. The saliva was stored in a proper container for PCR (Ependorf).

We carried out tests to detect the VZV DNA by means of the traditional PCR using the VVZ-7 (5′ ATG TCC GTA CAA CAT CAA CT 3′/5′CGA TTT TCC AAG AGA GAC GC 3′) primers previously described for this virus.

## RESULS

We assessed 171 patients with PFP from August of 2002 to October of 2007. Of these, 120 fit the pre-established inclusion and exclusion criteria.

Of the 120 patients in the study, 64 (53.3%) were females and 56 (46.7%) were males. Age varied between three and 80 years, with a mean value of 37.55 (±17.36) years.

VZV was studied through the PCR technique, and it was found in two patients with BP (1.7%, with a prevalence confidence interval ranging between 0.2% and 5.9%).

In the control group we did not find VZV DNA in any of the samples.

This difference regarding PCR positiveness between the cases and controls, although present was not statistically significant, as shown by the Fisher's exact test p= 0.734 (Table 1).

**Table 1:** Results from the viral DNA in VZV, through the PCR technique, in saliva from 120 patients with Bell's palsy and 20 control cases.

VZV			Total
	PCR positive	PCR negative	
BP	2	118	120
Controls	0	20	20
Total	2	138	140

BP = Bell's Palsy VZV = Varicella Zoster Virus PCR = Polymerase chain reaction

### Description of the PCR positive cases for VZV

1) Male, 62 years old with PFP on the left side, one day after hospital admission. The palsy degree at this time was HB VI. In this first assessment, saliva was collected from the patients, where the VZV was detected. The patient no longer returned to our clinic for future visits.
2) Male, 62 years old, with right side PFP, 12 hours after hospital admission. The palsy degree in the first visit was HB IV. We collected saliva from the patient in this first visit, from where we detected the VZV. The patient returned after one week, maintaining the same level of palsy and we chose to do a new saliva collection in order to confirm the results found. This second sample also showed VZV DNA. After four weeks the patient returned and we noticed palsy level HB III.

## DISCUSSION

We found two patients with positive PCR for VHS. Although such result is not statistically significant, two factors lead us to believe that there could be an etiological relation associated with these findings.

First of all, no control group sample was positive. Quoting Murakami et al.^29^: “the positive result means only that the virus is present, which leads us to believe in reactivation because of the fact that the controls were all negative”.

In second, one of the patients had two positive results for VZV. This tells us that the positiveness was not random or the result of contamination, suggesting an association of the PFP to the virus presence in the saliva.

Compared to findings from the international literature, the frequency of the VZV found in BP patients in our population was considered low.

Furuta et al.^10^ found six VZV-positive patients in the PCR from the oropharynx swab of 36 BP patients. The authors collected from one to four samples per individual, and they did not explain the criteria used to choose the number of samples. It may be that since they collected more samples from each patient, they had more positive findings.

This same Japanese group^19^ described VZV positiveness through PCR in 23 of the 26 patients (88%) who had BP and were seronegative for VHS. In the initial sample of patients with BP, 82% of the patients had serological alterations regarding VHS; thus, this study was held with a restricted number of individuals with BP (18% only).

Furuta et al.^19^ and Kawaguchi et al.^30^ found greater positiveness for VZV with the use of serology, if compared to the PCR. We would need studies comparing the two techniques for VZV in patients with BP.

The results from the present study are different from the ones found by Pitkäranta et al.^12^, who showed 10% of VZV positiveness. Nonetheless, the authors used tears, making it even more difficult to compare the studies.

Furuta et al.^31^, quantified the VZV copies and noticed that the viral load had a peak close to the day of the skin lesions manifestation. It is suggested that some negative PCR results for VZV could correspond to the drop in viral load.

Furuta et al.^32^, observed patients with SRH and described a patient with only one small oropharyngeal lesion who was not VZV-positive. We could argue that the lower the disease manifestation, the lower is the positiveness found in the PCR. In the present paper, since all the patients with some manifestation of the herpes disease were taken off the study, we would expect to find a lower positiveness, as it was the case.

All frequency differences may have happened because of the different population characteristics, which include both a racial or biological predisposition, as well as an association with the medium.

These data corroborate the Idea that the population, geographic and cultural differences can lead to different epidemiological manifestations of BP; and it is necessary to carry out regional studies.

It is known that there is great difficulty in establishing the pros and cons of using antiviral drugs. Two of the main studies which assess the efficacy of antiviral drugs, one of them is Japanese^33^ and the other one is En-glish^34^, yielded opposite results. This difference may have happened thanks to the diversity of factors which characterize the populations studied, which makes it difficult to establish a global treatment approach.

The present study shows that the viral reactivation frequencies are still not studied enough in the West. The goal of describing these incidences in our settings was to check the importance of the viruses studied in our population, so that based on these numbers we can establish better assessment and treatment protocols for our population of patients with BP.

## CONCLUSION

We noticed a frequency of 1.7% of VZV in saliva samples of patients with Bell's palsy by means of the PCR technique.
